# Supplementary material for: Predicting adverse pregnancy outcome in Rwanda using machine learning techniques
Source: PLoS One. 2024 Dec 5;19(12):e0312447. doi: 10.1371/journal.pone.0312447 (PMC11620651; doi:10.1371/journal.pone.0312447)
Supplement: Attachment — Submitted filename: A rebuttal Letter - reviewers.pdf [file pone.0312447.s003.pdf]

Editorial Office

PLOS ONE

Dear Sir/Madam

Thank you for the opportunity to revise and resubmit our manuscript titled "Predicting Adverse Pregnancy Outcome in Rwanda Using Machine Learning Techniques" (Manuscript ID: PONE-D-23-34183). We appreciate the constructive feedback provided by the reviewers and have made the necessary revisions to address all concerns. Below, we provide a detailed point-by-point response to the reviewers' comments, outlining the amendments made to the manuscript.

Editor’s Comments

Follow PLOS ONE manuscript format:

Amendments: Headings were reformatted, tables and figures were referenced and placed according to PLOS ONE format. Supporting information was also added.

Reviewers Comments

- *The conclusions must be drawn appropriately based on the data presented:*

Amendments: The conclusion was revised to ensure it aligns with the results and clarifies how the data supports the findings.

- *PLOS Data Policy:*

Amendments: A data availability statement was added, indicating that the data are available at www.dhsprogram.com.

- *The manuscript must be intelligible and written in standard English:*

Amendments: Grammatical errors were addressed, and the language was revised for clarity. We will continue to refine the language throughout the manuscript.

- *Clarification of percentages in the abstract:*

Amendments: The sentence was revised to clarify that "The findings showed that 93.4% of pregnancies resulted in live births, while 4.5% ended in miscarriage, and 2.1% in stillbirth".

- *Clarification on "multiple unions" and antenatal care (ANC):*

Amendments: "Multiple unions" was clarified as referring to polyandry. We also clarified that both married and unmarried women were considered in the analysis of ANC visits.

- *Keywords should be at least five:*

Amendments: Keywords were updated to include: Predictive modeling, Survey, Adverse pregnancy outcomes, Machine learning, Rwanda.

- *Lengthy introduction and missing references:*

Amendments: The introduction was shortened, and references were added to support the statement regarding global maternal fatalities in 2017 in Sub-Saharan Africa and Southern Asia.

- *Restructure of introductory section and improvement of language:*

Amendments: The introduction was revised to improve clarity and logical flow. Language was also improved for better readability.

- *Clarification of pregnancy loss as an adverse outcome:*

Amendments: We revised the dependent variable section to explain that pregnancy loss, including stillbirths, miscarriages, and abortions, was considered adverse due to potential physical, emotional, and health consequences. We also acknowledged that unwanted pregnancies could lead to pregnancy losses that may not be perceived as adverse.

- *Inclusion of maternal mortality as an adverse outcome:*

Amendments: While maternal mortality is undoubtedly an adverse outcome, we clarified that our study focused specifically on fetal and infant-related pregnancy outcomes. Maternal mortality was not included as a dependent variable.

- *Clarification of age range and marital status in univariate analysis:*

Amendments: The age range (15-49) for reproductive women was explicitly stated, and we provided clarification regarding the marital status of the women analyzed.

- *Incorporating machine learning predictions and identifying high-risk categories:*

Amendments: We included a detailed explanation of the machine learning predictions, highlighting that advanced maternal age, multiple unions, delayed first sexual intercourse, lack of ANC visits, and unmarried status were the most significant risk factors identified by the KNN model.

- *Clarification on pregnancy loss, deliberate abortions, and study limitations:*

Amendments: The dependent variable section was revised to clarify that pregnancy loss included both stillbirths and abortions (induced or spontaneous). We acknowledged the inability to distinguish between wanted and unwanted pregnancies in the DHS data as a limitation.

- *Study limitations:*

Amendments: A detailed limitations section was added, addressing issues such as the cross-sectional nature of the study, the inability to distinguish between wanted and unwanted pregnancies, and the exclusion of maternal mortality.

- *Research, policy, and practice implications:*

Amendments: We added a section outlining the implications of the study for research, policy, and practice. Specifically, we emphasized the need for further research to validate machine learning models, the importance of expanding access to ANC services in policy, and the use of machine learning tools in clinical practice for improving pregnancy outcome prediction and intervention .

Thank you for your consideration.

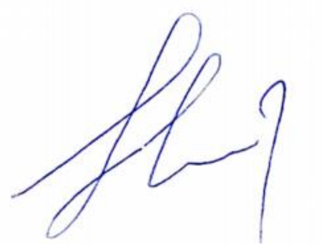

Sincerely,

Theogene Kubahoniyesu
