# Supplementary material for: Predicting adverse pregnancy outcome in Rwanda using machine learning techniques
Source: PLoS One. 2024 Dec 5;19(12):e0312447. doi: 10.1371/journal.pone.0312447 (PMC11620651; doi:10.1371/journal.pone.0312447)
Supplement: Attachment — Submitted filename: A rebuttal Letter.pdf [file pone.0312447.s005.pdf]

Date: October 5, 2024

Dear Editor and Reviewers,

PlOS ONE Journal

We would like to thank you for your thorough review of our manuscript (ID: PONE-D-23-34183R1), titled “Predicting Adverse Pregnancy Outcome in Rwanda Using Machine Learning Techniques”, submitted to PLOS ONE. We appreciate the constructive comments and suggestions, which have greatly helped us improve the quality of our work. Below, we provide a point-by-point response to each of the **reviewers'** and editor's comments, indicating the revisions made to the manuscript.

Reviewer 1:

1. Key words: Please add Neonatal mortality, Maternal mortality

Response: We have added "Neonatal mortality" and "Maternal mortality" to the keywords section.

Amendment made in: Abstract

2. Discussion: State the limitation of the study. Also, add the policy implication of the study.

Response: The limitations of the study are already discussed in a separate section. We have now added a paragraph discussing the policy implications of the study.

Amendment made in: Discussion

3. Contribution of the study to knowledge

Response: We have added a section elaborating on the study's contribution to knowledge, highlighting its significance in using machine learning for predicting pregnancy outcomes in Rwanda.

Amendment made in: Contribution of the study

4. Kindly proofread the work

Response: The manuscript has been thoroughly proofread and edited for clarity and correctness.

Amendment made in: Whole manuscript

Reviewer 2:

5. Lines 59-60: Please cite your source

Response: We have added the correct citation (WHO, 2024) for the statement regarding maternal mortality in developing countries.

Amendment made in: Introduction

6. Line 83: What are WHO-recommended methods?

Response: We have revised the paragraph to clearly outline WHO-recommended methods, including medical and surgical methods, with appropriate referencing.

Amendment made in: Introduction

7. Line 122: The strengths of ML over the traditional statistical methods that have predominantly been used may be a better justification.

Response: We have revised the paragraph to better explain the advantages of machine learning algorithms over traditional statistical methods, emphasizing their ability to handle complex, non-linear relationships and large datasets.

Amendment made in: Introduction

8. The re-categorization of the dependent variable makes sense now. However, since the variable “pregnancy outcome” does not exist in the DHS, it is advised that the authors specify how the variable was obtained.

Response: We have clarified that the "pregnancy outcome" variable was derived as a composite variable from multiple existing variables in the RDHS dataset. We also specified that the analysis focused on pregnancies reported in the five years preceding the survey.

Amendment made in: Materials and Methods

9. Readers may wonder what 'advanced maternal age' means. Please describe your variables or provide citations if you adopted existing measurements.

Response: We have defined "advanced maternal age" as women aged 35 years and older and revised the manuscript to clarify other variables that may be unclear.

Amendment made in: Whole manuscript

10. "Model was trained with 20% and tested with the remaining 80% of the data" – This seems to be a mistake.

Response: This was indeed a typographical error. The correct data split was 80% for training and 20% for testing. We have corrected this in the manuscript.

11. The results show that delayed sexual debut is associated with adverse pregnancy outcomes, which contrasts with existing literature. Please explain this finding.

Response: We have added a paragraph in the discussion that explains this finding in the context of Rwanda, where delayed sexual debut may be associated with older maternal age at first pregnancy, which increases the risk of complications. We also discuss the implications of this finding on abstinence advocacy.

Amendment made in: Discussion

Editor's Comment:

1. Complete references

Response: We have ensured that all references are complete and added DOI and URL links where necessary.

Amendment made in: References

We believe that the revised manuscript now adequately addresses all the concerns raised by the reviewers and the editor. We are grateful for the insightful feedback provided and hope that the changes meet your expectations.

Thank you again for your time and consideration.

Sincerely,

Theogene Kubahoniyesu

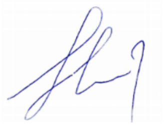

Corresponding Author
