# Supplementary material for: Predicting adverse pregnancy outcome in Rwanda using machine learning techniques
Source: PLoS One. 2024 Dec 5;19(12):e0312447. doi: 10.1371/journal.pone.0312447 (PMC11620651; doi:10.1371/journal.pone.0312447)
Supplement: Attachment — Submitted filename: Review of the Paper_13August2024.docx [file pone.0312447.s001.docx]

**Review of the Paper: The use of machine learning (ML) to predict adverse pregnancy outcomes in Rwanda**

The paper is timely and relevant, particularly in the context of improving maternal and neonatal health outcomes. The abstract could benefit from a brief mention of the significance or novelty of using ML in this specific context, which would help to frame the study's contribution to the field more effectively.

**Introduction:**

The introduction is somewhat lengthy and could be streamlined to focus more on the research gap and the rationale for using ML.

**Methods:**

The explanation of ML techniques could be enhanced with more details on the specific parameters used for each algorithm. Additionally, the rationale for choosing the K-nearest neighbours (KNN) model as the best performer should be further elaborated.

**Results:**

The results section could benefit from more detailed comparisons of the performance of different ML models. Including a table summarizing the performance metrics for all models would make it easier for readers to compare them.

**Discussion:**

The discussion could be strengthened by addressing the limitations of the study, particularly the use of secondary data and potential biases inherent in the data collection process. The implications for policy and practice could also be more explicitly discussed.

**Conclusion:**

The conclusion could be more impactful by including specific recommendations for future research or practical applications of the findings.

**Overall:**

Is it Publishable? The paper is largely well-written and covers a relevant and timely topic, making it a strong candidate for publication. However, it would benefit from revisions that enhance clarity, conciseness, and depth in certain sections, particularly the introduction, discussion, and results. The novelty and contribution of the study could be highlighted more effectively, and the methodological rigor could be emphasized with additional details on the ML models used. Addressing these areas would likely improve the paper's chances of acceptance in a peer-reviewed journal.
