# Supplementary material for: Predicting adverse pregnancy outcome in Rwanda using machine learning techniques
Source: PLoS One. 2024 Dec 5;19(12):e0312447. doi: 10.1371/journal.pone.0312447 (PMC11620651; doi:10.1371/journal.pone.0312447)
Supplement: Attachment — Submitted filename: comments.docx [file pone.0312447.s002.docx]

Direct quotes from the paper are in red

Abstract

The first section is rather called ‘background’ than ‘objective’. The statement “Adverse pregnancy outcomes have significant effects on both the mother and the infant” is confusing because adverse outcome is itself an effect. You need to focus on informing readers how leveraging on the predictive power of ML models to foretell adverse outcomes could inform interventions aimed at prevention. ML is central to your work…its preferential use over statistical models should be prominent.

Results:

The KNN was the most effective model for predicting adverse pregnancy outcomes…..then where are the predictions from KNN?

Your results that “Advanced maternal age (AOR: 3.452, 95% CI: 1.946-6.680), a higher age at first sexual intercourse (AOR: 1.421, 95% CI: 1.300 – 1.611), and living with more than one union (AOR: 1.320, 95% CI: 1.104 – 1.573) were risk factors for adverse pregnancy outcomes. However, the risk was lower among married women (AOR: 0.894, 95% CI: 0.787 – 0.966) and mothers who attended antenatal care (ANC) visits (AOR: 0.801, 95% CI: 0.664 – 0.9615)” are from statistical regression…where are your ML predictions? Which categories of women are most prone to adverse outcomes, based on ML models?

“Conclusions: ML algorithms can be used for accurately identifying potential risks at an early stage of pregnancy to enable healthcare professionals to intervene promptly and implement appropriate interventions and treatments” but the results are not here. You have only shown the risk factors for adverse outcomes as regressions would. It would have been understandable if regression was your highest performing model.

Main Body

Materials and information used in the background needs to be cited and well referenced. The background will greatly benefit from technical edits.

What have other authors reported about the risk factors for adverse pregnancies? How is this study superior or providing new knowledge? The authors may achieve these by providing justification for their use of machine learning…

Methods

“The women's dataset on 14634 women was used”

Did the study use all the data in the 2019/2020 RDHS, including women with no sex experience? What is the total sample? Which sub-groups were removed based on inclusion and exclusion criteria? If none, the authors need to state all these.

Description of variables

“Dependent variable Pregnancy outcome has different types, these include live birth (full term or preterm birth), stillbirths, miscarriage, both induced and spontaneous abortion. The dependent variable for this study was pregnancy outcome with two classes; live birth and pregnancy loss.”

The paper is not clear about what pregnancy loss means. Are you referring to both abortion and still birth as adverse outcome? If yes, what if the abortion was deliberate? As you may not be able to separate these in DHS, you may need to acknowledge them as limitations of your data.

Independent variables: The independent variables also referred to as predictors included the socio-demographic information and maternal characteristics of respondents.

You need to explain any modification made to the original DHS measure

“K-Fold cross validation (k=10) was used to split the training and test dataset” what % was used for train and test. These need to be specified for evaluation and reproducibility purpose.

Discussion

The results from our study indicated that during the five years preceding the Rwanda demographic and health survey of 2029-2020, 93.4% of the pregnancies resulted in live births, 4.5% ended in miscarriage while 2.1% ended in stillbirths. this conforms with the recent Rwanda demographic and health survey which showed that 21 in 1000 pregnancies end in stillbirths. the findings from our study are below the global estimate of miscarriage 20% which means that Rwanda continues to drop the pregnancy losses rate through improving maternal services

The results showed that Age of respondent was significantly associated with adverse pregnancy outcome. The more the age of respondent, the higher the risk of adverse pregnancy. Several studies have shown that advanced maternal age is associated with increased risk of adverse pregnancy outcomes such as preterm birth, low birth weight, and stillbirth.

What are the research, policy and practice implications of the study?

The authors did not acknowledge the limitations of the study.
