# Supplementary material for: Predicting adverse pregnancy outcome in Rwanda using machine learning techniques
Source: PLoS One. 2024 Dec 5;19(12):e0312447. doi: 10.1371/journal.pone.0312447 (PMC11620651; doi:10.1371/journal.pone.0312447)
Supplement: Attachment — Submitted filename: review_again.docx [file pone.0312447.s004.docx]

Direct quotes from the manuscript are in red

Thanks to the authors for taking their time to attend to the reviewers’ comments. However, it would have been expected that a point-by-point response to the comments would be uploaded, as is the standard, alongside the reviewed manuscript.

Introduction

Lines 59 - 60: please cite your source

Lines 73 – 74: really? How are pregnancy outcomes predictable by mothers? These are not in the ‘3’ you cited

Line 83: what are WHO-recommended methods?

Line 122: The strengths of ML over the traditional statistical methods that have predominantly been used may be a better justification.

Dependent variable

The re-categorization of the dependent variable makes sense now. However, since the variable “pregnancy outcome’ does not exist in the DHS, it is advised that the authors specify how the variable was obtained i.e. whether it is a composite gotten from a combination of other variables, an operationalized term from another variable in the DHS, or a simulation. Also, authors need to state the time reference, i.e. does this refer to the women’s pregnancy in the last one or five years?, This is important for open science (verifiability and reproducibility), which is the essence of the methodology section.

Independent variables

Authors need to provide descriptions of the independent variables. For instance, the results show that *“…woman with 48 advanced maternal age, those in multiple unions, and those who delayed their first sexual intercourse, who lacked adequate ANC were more prone to adverse pregnancy outcomes…*”

Readers may wonder what ‘advanced maternal age/ means? Please describe your variables or provide citations if you adopted existing measurements. Please do this for variables that are not straight forward.

Machine learning algorithms

*“Model was trained with 20% and tested with the remaining 80% of the data”*

Really? I guess this was a mistake. Premising prediction on 20% training must only have given spurious results. Training must take at least 50%, even up to 80% in many cases.

Discussion

The results show that “The KNN model highlighted that women with advanced maternal age, those in multiple unions, 349 and those who delayed their first sexual intercourse were more prone to adverse pregnancy 350 outcomes”

There is overwhelming evidence in the literature that delaying first sexual intercourse (unlike exposure before age 18) is associated with positive maternal outcomes. If the result of this study shows otherwise, authors need to spend time explaining why…and the implications of this on abstinence advocacy.

Please attach a point-by-point response If this manuscript is sent back to me for review.
